# A Parameter Self-Calibration Method for GNSS/INS Deeply Coupled Navigation Systems in Highly Dynamic Environments

**DOI:** 10.3390/s18072341

**Published:** 2018-07-18

**Authors:** Zang Chen, Jizhou Lai, Jianye Liu, Rongbing Li, Guotian Ji

**Affiliations:** 1 College of Automation Engineering, Nanjing University of Aeronautics & Astronautics, Nanjing 210016, China; chenzang@nuaa.edu.cn (Z.C.); ljyac@nuaa.edu.cn (J.L.); lrbing@nuaa.edu.cn (R.L.); nuaajgt@nuaa.edu.cn (G.J.); 2 Jiangsu Key Laboratory of Internet of Things and Control Technologies, Nanjing University of Aeronautics & Astronautics, Nanjing 211106, China

**Keywords:** deeply coupled, highly dynamic environments, norm, parameter self-calibration

## Abstract

The GNSS/INS (Global Navigation Satellite System/Inertial Navigation System) navigation system has been widely discussed in recent years. Because of the unique INS-aided loop structure, the deeply coupled system performs very well in highly dynamic environments. In practice, vehicle maneuvering has a big influence on the performance of IMUs (Inertial Measurement Unit), and determining whether the selected IMUs and receiver parameters satisfy the loop dynamic requirement is still a critical problem for deeply coupled systems. Aiming at this, a new parameter self-calibration method based on the norm principle is proposed which explains the relationship between IMU precision and the velocity error of the system; the method will also provide a detailed solution to calculate the loop steady-state tracking error, so it will eventually make a judgment about the stability of the tracking loop under present system parameter settings. Lastly, a full digital simulation platform is set up, and the results of simulations show good agreement with the proposed method.

## 1. Introduction

GNSS/INS deeply coupled technology is an important branch of current research in the field of integrated navigation systems. The theory around the loop assistance mechanism and the navigation performance improvement is quite mature, and the deeply coupled system has been applied in the field already.

The deeply coupled system introduces the inertial information into the tracking loop of the receiver, which means that the dynamic stress on the receiver could be highly reduced. Hence, this kind of system performs very well in highly dynamic environments. Around this topic, many research institutions and scholars have been working hard to improve the system performance. In terms of loop theory research, Zhang T. proposed an error analysis method to make the connection between most error sources and the PLL (Phase Lock Loop) tracking loop in 2015 [1]. Liu G. studied the relationship between MEMS (Micro Electro Mechanical Systems) IMU precision and tracking loop stability in 2013, and the bias stability of the IMUs was proposed from the angle of usability [2]. Zeng Q. studied the loop control scheme between loop measurements and INS navigation data in 2016, and two control schemes were derived which were proved to be correct [3]. In 2014, Kirkko-Jaakkola studied the jamming mitigation performance of a deeply coupled GNSS-INS system with a low-cost MEMS IMU and gave actual experimental results instead of simulations [4]. Adeel developed a good structure for a tracking loop containing a multicarrier VPLL (Vector PLL) in 2015, and the performance of the proposed architecture was validated by means of tests under different conditions [5].

Moreover, the implementation of actual deeply coupled systems has been under development. In 2014, KIT (Karlsruhe Institute of Technology) set up a highly dynamic and deeply coupled verification prototype; a Simulink software receiver was used to complete the algorithm verification on the Matlab platform for the first time, and a real-time solution for deeply coupled architecture was achieved [6]. In 2015, the Rockwell Collins Company launched the NavFire-E Series of high-performance integrated navigation systems, which represent the highest standards of the current practical applications. The highly integrated micro-inertial and anti-jamming receiver technology were used to achieve high output accuracy and high reliability of the system [7].

However, the performance of inertial devices will be affected seriously by highly dynamic environments. The key point is to make sure that the assistance information used to reduce the dynamic stress is reliable. How to select correct inertial devices and receiver parameters to satisfy the loop dynamic requirement is still a problem for the system. However, quite a few articles have studied this: Liu G. discussed the usability of a MEMS IMU in a deeply coupled system, but the g-sensitivity error of gyroscope under high dynamic environments was ignored [2]; Kirkko-Jaakkola built an actual experimental platform with a MEMS IMU, but it was not clear how to select the appropriate IMU devices [4]. In this paper, a mathematic model of the deeply coupled system including the g-sensitivity error of gyroscope is introduced. Then, according to the norm principle, the error propagation properties are analyzed by mathematical deduction and this process gives the quantitative velocity error caused by IMU error. In addition, a new method which combines with the tracking error model to determine the loop stability is introduced. It will eventually make a judgment about the stability of the tracking loop under certain parameter settings. Finally, the correctness of the proposed method was verified by several simulations based on a digital simulation platform.

## 2. Mathematical Model of a Deeply Coupled Navigation System

**Figure 1:**
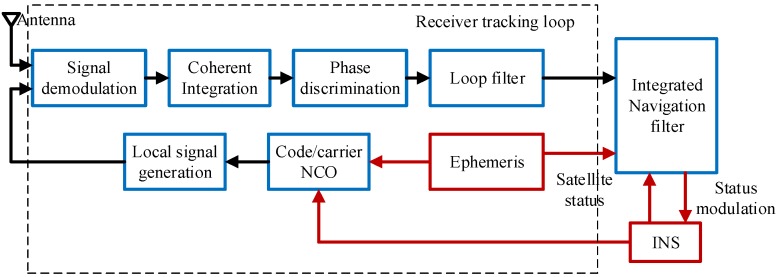
Structure diagram of the GNSS/INS deeply coupled system.

The core concept of the deeply coupled system aided by an IMU is to extract the doppler frequency and code phase deviation based on the inertial information and satellite ephemeris, and this can help to control the code/carrier NCO (Numerically Controlled Oscillator) in the tracking loop. This structure can ensure the system maintains high performance of the tracking loop in narrow bandwidth conditions. The overall architecture of the system is shown in Figure 1.

The performance of the inertial devices is obviously affected by highly dynamic circumstances, so the IMUs’ error model should be established first. Due to the smaller and lighter tendency of the deeply coupled system, this article mainly discusses the MEMS device.

The error model of gyroscope and accelerometer are given as follows:

(1) $${\tilde{\omega }}_{ib}^{b}=\omega_{ib}^{b}+b_{g}+g_{s}f_{ib}^{b}+\eta_{g},$$

(2) $${\tilde{f}}_{ib}^{b}=f_{ib}^{b}+b_{a},$$

where ${\tilde{\omega }}_{ib}^{b}$ is the angular velocity measurement of the gyroscope, i denotes the inertial coordinate system, b denotes the body coordinate system, $b_{g}$ is the gyroscope bias, $g_{s}$ is the matrix of g-sensitivity coefficients, $f_{ib}^{b}$ is the specific force, $\eta_{g}$ is the measurement error of gyroscope, ${\tilde{f}}_{ib}^{b}$ is the specific force measurement of the accelerometer, and $b_{a}$ is the accelerometer bias [8,9].

In order to analyze the relationship between the IMU errors and the INS navigation errors in the deeply coupled system, the state variables related to the inertial system in the integrated navigation filter could be expressed as follows:

(3) $$X={\left[\begin{array}{cccccc} \delta R^{e} & \delta V^{e} & \delta \psi^{e} & \delta \omega^{b} & \delta A^{b} & \delta T \end{array}\right]}^{T},$$

where $\delta R^{e}$ are the error vectors of the 3D position, $\delta V^{e}$ are the error vectors of 3D velocity, and $\delta \psi^{e}$ are the error vectors of 3D attitude; e denotes the ECEF coordinate system; $\delta \omega^{b}$ are the error vectors of the gyroscopes; and $\delta A^{b}$ are the error vectors of the accelerometers in the body coordinate system. δT are the clock error vectors including clock bias error $\delta T_{bias}$ and clock drift error $\delta T_{drift}$ [10,11].

The equation of state is given as follows:

(4) $$\left[\begin{array}{c} \delta {\dot{R}}^{e}\\ \delta {\dot{V}}^{e}\\ \delta {\dot{\psi }}^{e}\\ \delta {\dot{\omega }}^{b}\\ \delta {\dot{A}}^{b}\\ \delta \dot{T} \end{array}\right]=\left[\begin{array}{cccccc} 0 & I & 0 & 0 & 0 & 0\\ G-{\left({\tilde{\Omega }}^{e}\right)}^{2} & -2{\tilde{\Omega }}^{e} & {\tilde{F}}^{e} & 0 & C_{b}^{e} & 0\\ 0 & 0 & -{\tilde{\Omega }}^{e} & C_{b}^{e} & 0 & 0\\ 0 & 0 & 0 & 0 & 0 & 0\\ 0 & 0 & 0 & 0 & 0 & 0\\ 0 & 0 & 0 & 0 & 0 & \Lambda \end{array}\right]\left[\begin{array}{c} \delta R^{e}\\ \delta V^{e}\\ \delta \psi^{e}\\ \delta \omega^{b}\\ \delta A^{b}\\ \delta T \end{array}\right]+\left[\begin{array}{c} 0\\ 0\\ 0\\ \eta_{g}\\ b_{a}\\ \omega_{T} \end{array}\right],$$

(5) $$G=\frac{\mu }{r^{3}}\left[\begin{array}{ccc} -1+\frac{3x^{2}}{r^{2}} & \frac{3xy}{r^{2}} & \frac{3xz}{r^{2}}\\ \frac{3xy}{r^{2}} & -1+\frac{3y^{2}}{r^{2}} & \frac{3yz}{r^{2}}\\ \frac{3xz}{r^{2}} & \frac{3yz}{r^{2}} & -1+\frac{3z^{2}}{r^{2}} \end{array}\right],$$

(6) $${\tilde{\Omega }}^{e}=\left[\begin{array}{ccc} 0 & \omega_{e} & 0\\ -\omega_{e} & 0 & 0\\ 0 & 0 & 0 \end{array}\right]\;{\tilde{F}}^{e}=\left[\begin{array}{ccc} 0 & f_{z} & -f_{y}\\ -f_{z} & 0 & f_{x}\\ f_{y} & -f_{x} & 0 \end{array}\right]\;\Lambda =\left[\begin{array}{cc} 0 & 1\\ 0 & 0 \end{array}\right]\;$$

where G denotes the parameter of gravity, $\mu =3.986005\times 10^{14}(m^{3}/s^{2})$, which represents the product of the gravitation constant and the mass of earth; $\left[\begin{array}{ccc} x & y & z \end{array}\right]$ denote the position vectors in ECEF coordinates, $r=\sqrt{x^{2}+y^{2}+z^{2}}$, which is the distance to the center of the Earth; ${\tilde{\Omega }}^{e}$ is the matrix of the Earth’s rotational angular velocity, $\omega_{e}=7.292115\times 10^{-5}(rad/s)$; ${\tilde{F}}^{e}$ is the matrix of specific force in the ECEF coordinate system; $C_{b}^{e}$ is the direction cosine matrix between the body coordinate system and the ECEF coordinate system; Λ is the clock error coefficient matrix; $\omega_{T}={\left[0\;\omega_{f}\right]}^{T}$ is the clock error vectors; and $\omega_{f}$ is the clock drift error vectors.

To make sure that the system stability could be analyzed, Wagner J. F. studied the observability of the integrated system [12]. According to his conclusion, when the system consists of three antennas which form a triangle of sufficient area, observability would be guaranteed under all circumstances. Thus, this paper continues on the premise that the observability of the system is guaranteed by the system hardware.

## 3. Parameter Self-Calibration Method for Loops in Deeply Coupled Systems Based on Norm Analysis

According to the analysis in the above section, the position and velocity of the vehicle calculated by INS are directly involved in the feedback to the receiver tracking loop, and the carrier frequency error is mainly related to the velocity error, while the code phase error is only related to the position error. When the closed-loop Kalman filter is run to the steady state, the estimation error is convergent, and the accuracy of the filtering result is mainly determined by the covariance matrix of the state noise. Therefore, what needs to be considered is the INS error in one calculation cycle. Because the code phase error caused by the position error is much smaller than the carrier frequency error and leads to a quite small influence on the system, this article focuses on the discussion of the INS velocity error analysis.

### 3.1. Norm Analysis to the IMU Error Propagation Properties

Before the norm analysis referring to the INS error, the definition of the two kinds of norms should be made clear [13]. If A is a vector, the Euclid norm, or 2-norm, of A is ${\left\|A\right\|}_{2}=(\sum_{i=1}^{n}{\left|A_{i}\right|}^{2}{)}^{1/2}$. If B is a matrix, the Frobenius norm, or *F*-norm, of B is ${\left\|B\right\|}_{F}=(\sum_{i=1}^{m}\sum_{j=1}^{n}{\left|B_{ij}\right|}^{2}{)}^{1/2}$. Both of these two norms represent the meaning of spatial distance and satisfy the following axioms:

(1) ‖X‖≥0; further, ‖X‖=0 if, and only if, X=0. (Positive-definiteness)
(2) ‖aX‖=|a|‖X‖ for any scalar a. (Homogeneity)
(3) ‖X+Y‖≤‖X‖+‖Y‖. (Triangle inequality)

The constant coefficients theorem is described as follows: Let A be an matrix of m×m scalars, X be an m×n matrix of unknowns, and B be an m×n matrix of given functions. Then the differential equation

(7) $$\dot{X}=A\cdot X+B,$$

with initial value $X(t_{0})=X_{0}$ has the solution

(8) $$X(t)=e^{(t-t_{0})A}X_{0}+\int_{t_{0}}^{t}e^{(t-t_{0})A}B(\tau )d\tau ,$$

where $t_{0}$ denotes the initial moment.

According to the theorem above, Equation (4) could be expressed briefly as the following differential equation:

(9) $$\dot{X}=F\cdot X+W,$$

and the approximate analytical solution of Equation (7) is

(10) $$X(t_{0}+\Delta T)=e^{\Delta TF}X_{0}+\int_{t_{0}}^{t}e^{\Delta TF}Wd\tau ,$$

where ΔT denotes the time interval between two consecutive estimations.

From Equation (4), the velocity and position differential equation could be expressed as follows:

(11) $$\delta {\dot{R}}^{e}=\delta V^{e},$$

(12) $$\delta {\dot{V}}^{e}=[G-{\left({\tilde{\Omega }}^{e}\right)}^{2}]\delta R^{e}-2{\tilde{\Omega }}^{e}\delta V^{e}+{\tilde{F}}^{e}\delta \psi^{e}+C_{b}^{e}\delta A^{b},$$

where $\delta {\dot{R}}^{e}$ and $\delta {\dot{V}}^{e}$ represent the derivatives of position and velocity error vectors, respectively.

From Equation (11), the position error $\delta R^{e}$ in one time interval should be $\delta V^{e}\Delta T$. Thus, Equation (12) could be expressed as follows:

(13) $$\delta {\dot{V}}^{e}=[(G-{\left({\tilde{\Omega }}^{e}\right)}^{2})\Delta T-2{\tilde{\Omega }}^{e}]\delta V^{e}+{\tilde{F}}^{e}\delta \psi^{e}+C_{b}^{e}\delta A^{b}.$$

Combining with Equation (10), the velocity error caused by the accelerometer error is

(14) $${\left(\delta V^{e}\right)}_{acc-bias}=\frac{1}{F^{*}}e^{F^{*}t}C_{b}^{e}\delta A^{b},$$

(15) $$F^{*}=(G-{\left({\tilde{\Omega }}^{e}\right)}^{2})\Delta T-2{\tilde{\Omega }}^{e}.$$

Thus, from Equation (14), the velocity error caused by the accelerometer error is

(16) $$\begin{array}{cc} {\left(\delta V^{e}\right)}_{acc-bias} & \approx \left[\Delta TI-\Delta T^{2}{\tilde{\Omega }}^{e}+\frac{\Delta T^{3}}{2}(G-{\left({\tilde{\Omega }}^{e}\right)}^{2})+\frac{\Delta T^{3}{\left((G-{\left({\tilde{\Omega }}^{e}\right)}^{2})\Delta T-2{\tilde{\Omega }}^{e}\right)}^{2}}{6}\right]C_{b}^{e}\delta A^{b}\\ & \approx \left[\Delta TI-\Delta T^{2}{\tilde{\Omega }}^{e}+\frac{\Delta T^{3}}{2}G+\frac{\Delta T^{3}{\left({\tilde{\Omega }}^{e}\right)}^{2}}{6}\right]C_{b}^{e}\delta A^{b} \end{array},$$

and it should be noted that the small quantities of high order ($\Delta T^{\geq 4}$) in this step have been ignored.

Both sides of Equation (16) could be expressed in the norm form as follows:

(17) $${\left\|{\left(\delta V^{e}\right)}_{acc-bias}\right\|}_{2}\approx {\left\|\left[\Delta TI-\Delta T^{2}{\tilde{\Omega }}^{e}+\frac{\Delta T^{3}}{2}G+\frac{\Delta T^{3}{\left({\tilde{\Omega }}^{e}\right)}^{2}}{6}\right]C_{b}^{e}\delta A^{b}\right\|}_{2}.$$

According to the norm property, Equation (17) could be transformed into the following expression:

(18) $${\left\|{\left(\delta V^{e}\right)}_{acc-bias}\right\|}_{2}\leq \left[\Delta T+\Delta T^{2}{\left\|{\tilde{\Omega }}^{e}\right\|}_{F}+\frac{\Delta T^{3}}{2}{\left\|G\right\|}_{F}+\frac{\Delta T^{3}}{6}{\left\|{\left({\tilde{\Omega }}^{e}\right)}^{2}\right\|}_{F}\right]{\left\|C_{b}^{e}\right\|}_{F}{\left\|\delta A^{b}\right\|}_{2},$$

where $C_{b}^{e}$ is the unitary matrix, so ${\left\|C_{b}^{e}\right\|}_{F}=\sqrt{\left(tr(C_{e}^{b}C_{b}^{e})\right)}=\sqrt{3}$.

Equation (18) could be transformed into the following expression:

(19) $${\left\|{\left(\delta V^{e}\right)}_{acc-bias}\right\|}_{2}\leq \sqrt{3}\left[\Delta T+\sqrt{2}\omega_{e}\Delta T^{2}+\frac{\sqrt{6}\mu \Delta T^{3}}{2r^{2}}+\frac{\mu^{2}\Delta T^{3}}{r^{4}}\right]{\left\|\delta A^{b}\right\|}_{2},$$

because ${\left\|{\tilde{\Omega }}^{e}\right\|}_{F}=\sqrt{2}\omega_{e}$ and ${\left\|G\right\|}_{F}=\frac{\sqrt{6}\mu }{r^{2}}$, according to the definition of F-norm.

Also according to Equation (4), the velocity error caused by the gyro error could be concluded as follows with the same analysis idea above:

(20) $$\begin{array}{cc} {\left(\delta V^{e}\right)}_{gyro-bias} & =\frac{e^{-{\tilde{\Omega }}^{e}\Delta T}\Delta T}{-{\tilde{\Omega }}^{e}}{\tilde{F}}^{e}C_{b}^{e}\delta \omega^{b}\\ & \approx (\Delta T^{2}-\frac{\Delta T^{3}{\tilde{\Omega }}^{e}}{2}){\tilde{F}}^{e}C_{b}^{e}\delta \omega^{b} \end{array},$$

where both sides of Equation (20) could be expressed in the norm form as follows:

(21) $$\begin{array}{cc} {\left\|{\left(\delta V^{e}\right)}_{gyro-bias}\right\|}_{2} & \approx {\left\|(\Delta T^{2}-\frac{\Delta T^{3}{\tilde{\Omega }}^{e}}{2}){\tilde{F}}^{e}C_{b}^{e}\delta \omega^{b}\right\|}_{2}\\ & =(\Delta T^{2}+\frac{\Delta T^{3}}{2}{\left\|{\tilde{\Omega }}^{e}\right\|}_{F}){\left\|{\tilde{F}}^{e}\right\|}_{F}{\left\|C_{b}^{e}\right\|}_{F}{\left\|\delta \omega^{b}\right\|}_{2}\\ & \leq \sqrt{6(f_{x}^{2}+f_{y}^{2}+f_{z}^{2})}(\Delta T^{2}+\frac{\sqrt{2}\omega_{e}\Delta T^{3}}{2}){\left\|\delta \omega^{b}\right\|}_{2} \end{array}.$$

From Equations (19) and (21), the relationship between the IMU errors and the related velocity error is clear. It could be concluded that the INS velocity error mainly depends on four factors: the gyro error $\delta \omega^{b}$, the accelerometer error $\delta A^{b}$, the time interval ΔT, and the vehicle maneuvering which can be expressed as $\sqrt{f_{x}^{2}+f_{y}^{2}+f_{z}^{2}}$.

Through the mathematical deduction above, the quantitative velocity error can be calculated directly when the IMUs’ precision and the dynamic requirement for the system are determined. This will do great favors for the stability judgement of the tracking loop in the next step.

### 3.2. Parameter Self-Calibration Method for the Tracking Loop in a Deeply Coupled System

A method based on the norm property was proposed to analyze the relationship between the IMU errors and the system velocity error above; the next step is to make the judgement on the stability of the tracking loop in deeply coupled systems in highly dynamic environments.

For the deeply coupled system, the total tracking error of carrier loop could be given as follows:

(22) $$\sigma_{PLL}=\sqrt{{\sigma_{tPLL}}^{2}+{\sigma_{v}}^{2}+{\sigma_{A}}^{2}}+\frac{\theta_{e}}{3},$$

(23) $$\sigma_{tPLL}=\frac{180}{\pi }\sqrt{\frac{B_{L}}{C/N_{0}}\left[1+\frac{1}{2T_{coh}\cdot C/N_{0}}\right]},$$

(24) $$\sigma_{A}=360\frac{c}{\lambda }T_{coh}\sigma_{A}(\tau ),$$

(25) $$\theta_{e}=360\frac{{a_{n}}^{n}R^{\left(n\right)}}{\lambda B_{L}{}^{n}},$$

where $\sigma_{tPLL}$ is the standard deviation of phase error caused by the thermal noise, $\sigma_{v}$ is the standard deviation of phase error caused by the jitter of the reference oscillator frequency, $\sigma_{A}$ is the standard deviation of phase error caused by the frequency drift of the Allan crystal oscillator, and $\theta_{e}$ is the dynamic stress error caused by the relative movement.

Further, $B_{L}$ is the equivalent bandwidth of the loop, $C/N_{0}$ is the carrier-to-noise ratio, $T_{coh}$ is the coherent integration time, c is the speed of light, λ is the carrier wavelength, $\sigma_{A}(\tau )$ is the Allan standard deviation of the frequency, and $a_{n}$ is filter parameters of the loop; n is the loop order, $a_{1}=0.25,a_{2}=0.53,a_{3}=0.7845$ [8].

It needs to be emphasized that $R^{\left(n\right)}=\frac{d^{n}R}{dt^{n}}$, where $R^{\left(n\right)}$ is the *n*th-order derivative of the distance between the vehicle and the satellite.

Because the second-order locked loop is sensitive to the accelerated movement and the third-order loop is sensitive to the jerk movement, the velocity error should be transformed to the equivalent relative movement. Assuming that the INS-aided velocity error in one loop-aided cycle is $\delta v^{e}$, the (*n* − 1)th-order derivative is as follows:

(26) $${\left(\delta V^{e}\right)}^{\left(n-1\right)}=R^{\left(n\right)},$$

where $R^{\left(n\right)}$ represents the *n*th-order derivative of the position error vectors.

In order to keep the tracking loop stable, 3 times the variance of the phase measurement error should be less than 45° [14]. According to Equations (22), (25) and (26), the following inequation could be inferred:

(27) $$\sqrt{{\sigma_{tPLL}}^{2}+{\sigma_{v}}^{2}+{\sigma_{A}}^{2}}+120\frac{{a_{n}}^{n}{\left(\delta V^{e}\right)}^{\left(n-1\right)}}{\lambda {B_{L}}^{n}}\leq 15.$$

Furthermore, according to Equations (19) and (21), the (*n* − 1)th-order derivative of the INS-aided velocity error is

(28) $${\left(\delta V^{e}\right)}^{\left(n-1\right)}={\left\{\sqrt{3}\left[\Delta T+\sqrt{2}\omega_{e}\Delta T^{2}+\frac{\sqrt{6}\mu \Delta T^{3}}{2r^{2}}+\frac{\mu^{2}\Delta T^{3}}{r^{4}}\right]{\left\|\delta A^{b}\right\|}_{2}+\sqrt{6a^{2}}(\Delta T^{2}+\frac{\sqrt{2}\omega_{e}\Delta T^{3}}{2}){\left\|\delta \omega^{b}\right\|}_{2}\right\}}^{\left(n-1\right)},$$

where $\sigma_{v}$ is about 2°, n is the loop order, a is the sum acceleration of the vehicle, and ΔT is the time interval between two INS-aided moments.

So far, all the key parameters of the receiver are involved in Equations (27) and (28), and the whole deeply coupled system can be configured to satisfy the engineering requirement in consideration of the vehicle maneuvering. If the IMU precision related to $\delta A^{b}$/$\delta \omega^{b}$ and the dynamic requirement of the deeply coupled system related to a are defined, it can be directly determined whether the system can guarantee the stability of the tracking loop or not. The entire parameter self-calibration method is proposed above; the correctness and advantages will be proved next.

## 4. Highly Dynamic Simulations and Results

In order to prove the correctness and advantages of the proposed method, a full digital simulation platform based on Matlab was constructed. The orbital distribution and operation cycle of GPS (Global Position System) is generated according to actual GPS parameters; the IMUs’ information is selected from the data book of Analog Devices Inc. In this part, the norm-based analysis method is verified first, and after combining with three different kinds of MEMS gyros’ parameters, the actual result of the parameter self-calibration method is put forward.

### 4.1. Simulation Conditions and Track Settings

According to the data book of ADI Co., Ltd. product, three typical gyroscopes are selected; they are the high-precision gyroscope ADIS16490, the middle-precision gyroscope ADIS16448, and the low-precision gyroscope ADIS16300 (Analog Devices, Norwood, MA. USA). The main error parameters of the gyroscopes are shown in Table 1, and the bias errors of accelerometers are set as 16 mg/20 mg/60 mg.

**Figure 2:**
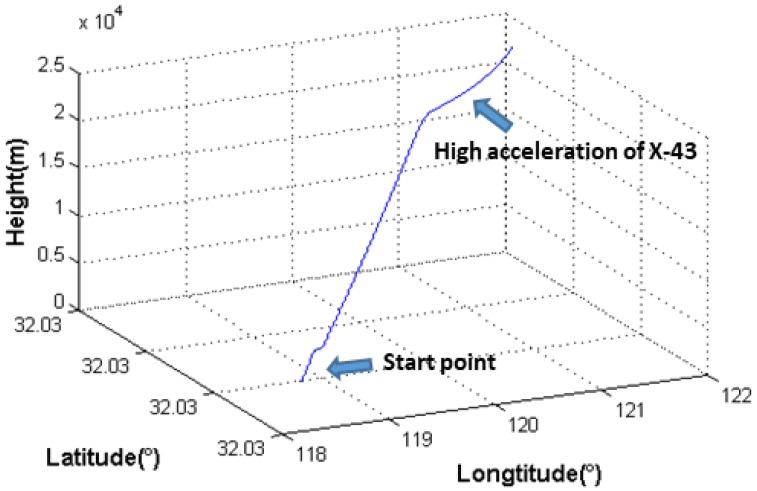
3D trajectory of the X-43 hypersonic aircraft.

The parameters of the GPS system are as follows: the carrier-to-noise ratio (CNR) is 50 dB-Hz, the bandwidth of the carrier tracking loop is 20 Hz, the coherent integration time is 1 ms, the Allan standard deviation of the frequency is 10^−6^ Hz, and the carrier wavelength of GPS L1 is 0.19 m. For the deeply coupled system, the calculation cycle of INS and the cycle of INS-aided loop control are both 10 ms, and the filter cycle time is 0.1 s. A typical dynamic trajectory of an X-43 hypersonic aircraft is set as the reference track from running take-off until level flight [15]. The initial position is in Nanjing where the coordinate is (18.80°, 32.03°, 10.00 m), the initial velocity is 0 m/s, and the initial heading angle is 90°. The 3D trajectory is as shown in Figure 2, and each stage of the dynamic trajectory is as shown in Table 2.

### 4.2. Simulation of Inertial Calculation

In this part, the correctness of the proposed norm analysis method will be proved from the aspect of inertial calculation. The calculated velocity error by norm analysis is compared with the Monte Carlo simulation result of the inertial calculation in one cycle.

#### 4.2.1. Norm Analysis Simulation

**Figure 3:**
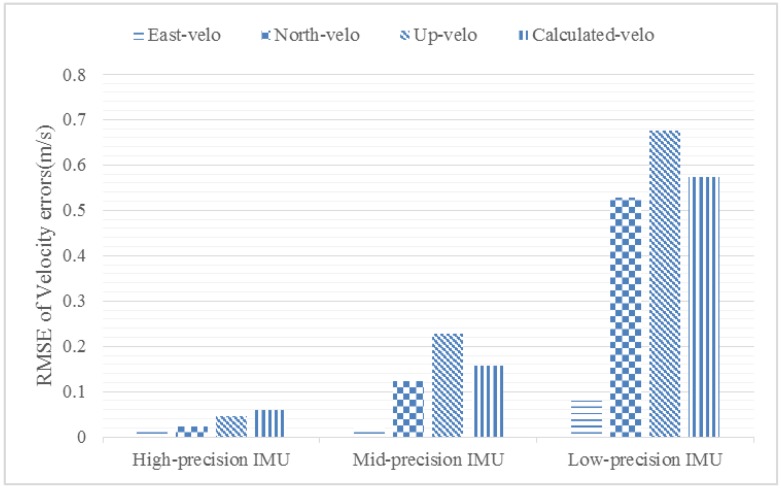
Histogram of root-mean-square errors (RMSEs) of velocity errors.

In order to prove the correctness of the norm analysis method above, the first 10 ms of the acceleration of the X-43 are picked up; these 10 ms give the greatest dynamic movement in the whole trajectory. The initial velocity of the vehicle is set as (2146.4 m/s, 0, 0). Ten Monte Carlo simulations were conducted and the root-mean-square errors (RMSEs) of three velocity errors were compared with the calculated velocity error from Equation (28). A histogram of the RMSE is shown in Figure 3, and the detailed statistical analysis of Figure 3 is shown in Table 3.

From Figure 3 and Table 3, the velocity error calculated by Equation (28) matches well with the RMSE of the velocity errors from the Monte Carlo simulation. Hence, the proposed norm analysis method is correct and the calculated velocity error can be trusted to work in the loop stability analysis. Further, the calculation times of the norm analysis method and the Monte Carlo method were compared by Matlab platform as performed on a PC equipped with an I7-7700 CPU; the result shows that the norm analysis method only takes 0.01 s to get the velocity error while the Monte Carlo method needs 0.42 s. So, from this part, we can see that the proposed norm analysis method is more concise, efficient, quantitative, and precise.

#### 4.2.2. Stability Simulation under Highly Dynamic Circumstances

With the help of the simulation above, the velocity error caused by the inertial calculation has been quantized, and the stability of the tracking loop is related to Equation (27) directly. So, in this part, the correctness of Equation (27) will be proved. 

**Figure 4:**
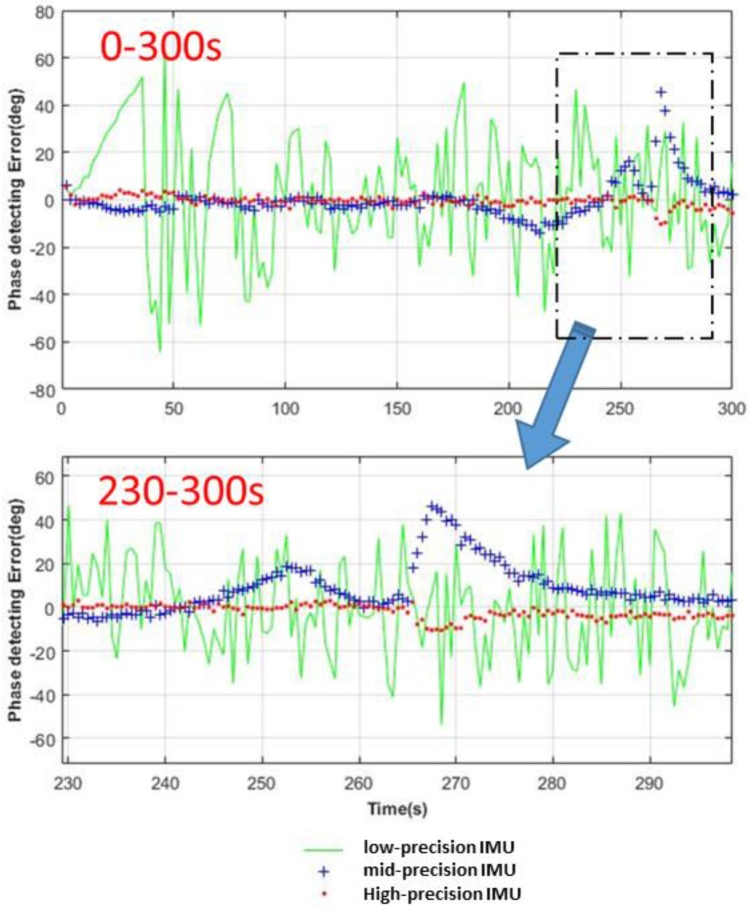
Comparison diagram of phase detecting error aided by three kinds of IMUs.

The parameters of both the INS and GNSS systems are set as the same as before; it should be mentioned that the Kalman Filter (KF) parameters should be configured according to the system and measurement parameters, and this article will not discuss the setting of KF parameters. The structure of the carrier loop is determined as the third-order phase-locked loop aided by the second-order frequency-locked loop, so n in Equation (27) is 3. The phase detection results of one picked tracking channel aided with three different precision IMUs are shown in Figure 4, and the phase errors calculated by the left-hand side of Equation (27) are shown in Table 4.

Figure 4 shows that the high-precision IMU helps maintain tracking through almost all the process. Only at the times of 265 s and 270 s does there exist a phase detecting error saltation, because the acceleration changed from 19 m/s/s to 300 m/s/s suddenly and the tracking loop cannot adjust to the great jerk instantly. During the 5 s acceleration of X-43, the high-precision IMU can help maintain tracking, and the middle-precision IMU performs worse than the high-precision IMU, but the low-precision IMU cannot guarantee the stability of the tracking loop even when the vehicle is under a low dynamic environment. Further, according to Table 4, the calculated phase errors show that both high-precision and middle-precision IMUs could satisfy the stability condition of Equation (27) but the low-precision IMU could not; this result agrees well with the phenomenon observed in Figure 4. 

**Figure 5:**
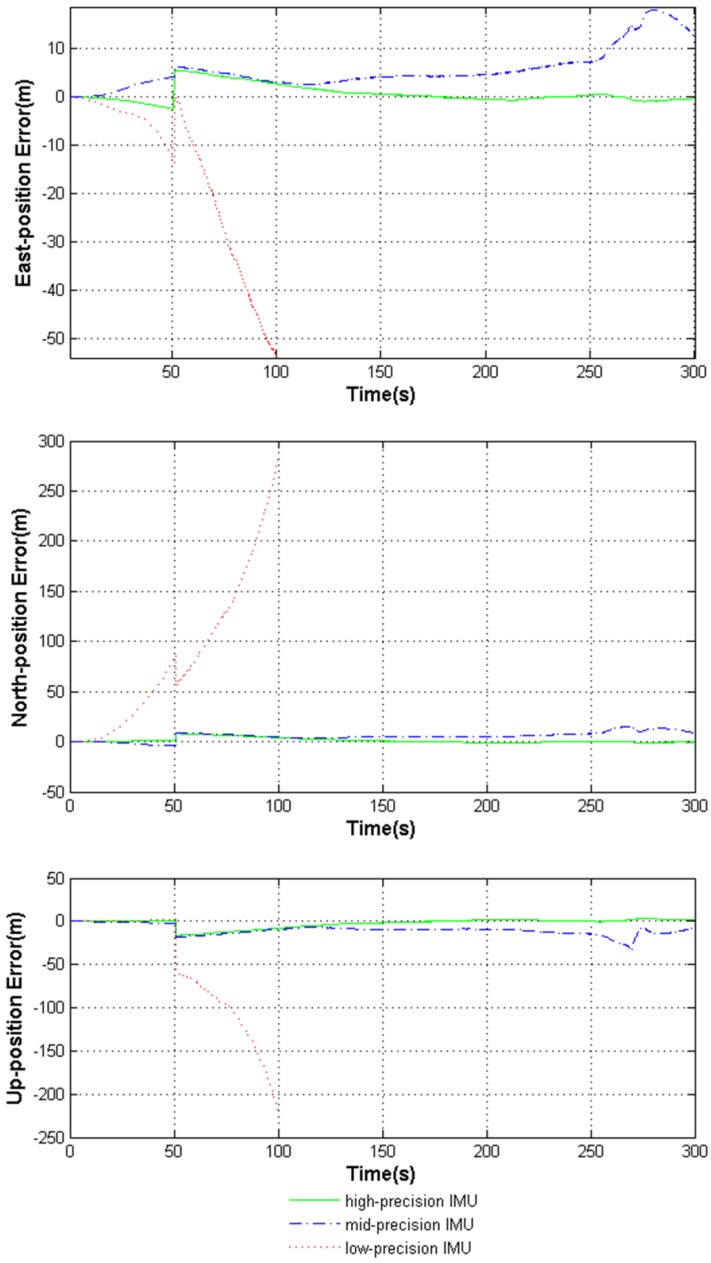
Comparison diagram of position error aided by the three kinds of IMUs.

**Figure 6:**
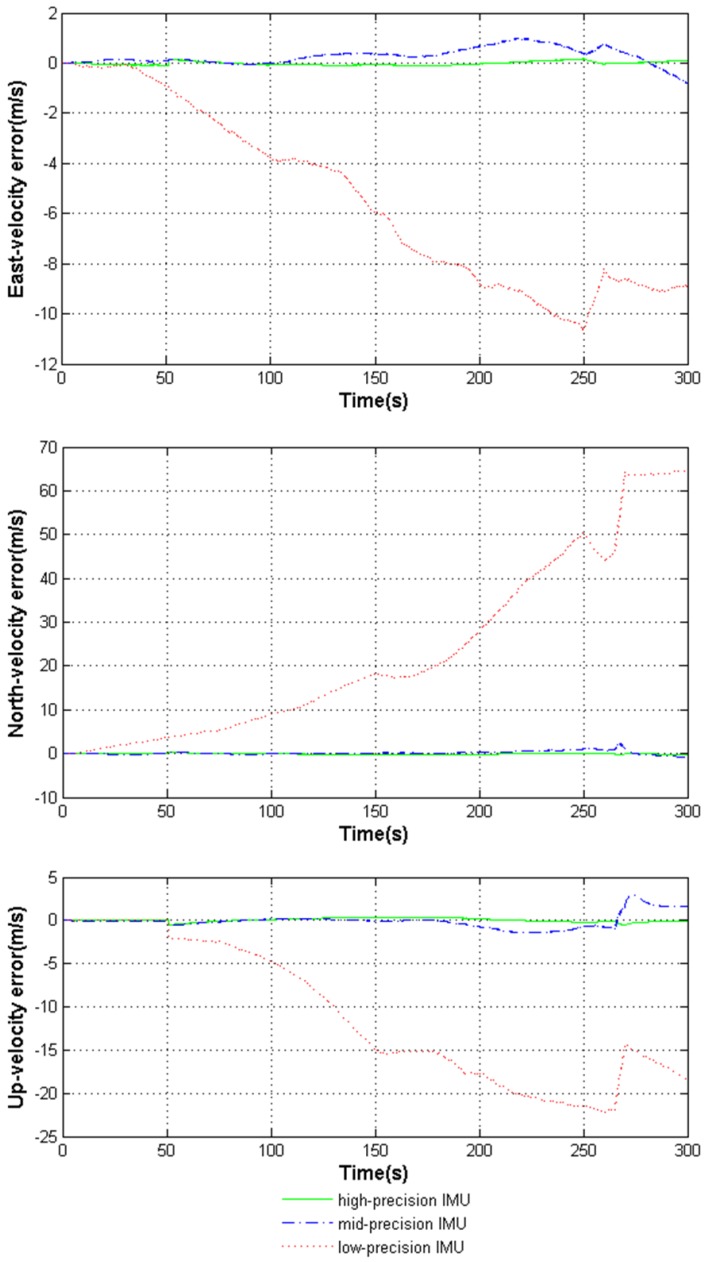
Comparison diagram of velocity error aided by the three kinds of IMUs.

The navigation results aided by the three kinds of IMUs are further compared in Figure 5 and Figure 6. The detailed statistical analysis of Figure 5 and Figure 6 is shown in Table 5.

Figure 5 and Figure 6 and Table 5 show that with the help of the high-precision IMU, the deeply coupled navigation system performs very well in highly dynamic environments. The middle-precision IMU can guarantee the navigation precision of the system but performs worse than the high-precision IMU. However, the low-precision IMU causes the system to lose positioning ability throughout the whole process.

In all, the simulation results above have proved that Equation (27) could make a correct judgment about the stability of the tracking loop in consideration of the IMUs’ precision, the system parameters, and the vehicle maneuvering.

## 5. Conclusions

A new mathematical model of a deeply coupled system including the g-sensitivity error of the gyroscope was proposed. According to the system model, the error propagation properties were analyzed by mathematical deduction from the perspective of the norm principle. Then, an inequality considering most of the deeply coupled navigation system parameters was proposed to judge the stability of the tracking loop. All the above theories were verified by a full digital simulation platform, and the simulation results proved the correctness of the relevant conclusions.

The proposed method can provide basic guidance for the design of deeply coupled systems. The stability judging process is reliable and flexible, and so can provide new ideas for the engineering realization of deeply coupled navigation systems. Also, the actual effect of the theory in practical systems will be checked in the near future.

## Figures and Tables

**Table 1:** Error parameters of three typical gyroscopes from ADI Co., Ltd.

Gyros ID	Bias Repeatability (°/s)	White Noise (°/s)	g-Sensitivity (°/s/g)	50 g-Sensitivity (°/s)
ADIS16490	0.05	0.05	0.005	0.25
ADIS16448	0.50	0.27	0.015	0.75
ADIS16300	2.00	1.10	0.050	2.50

**Table 2:** Parameters of the X-43 hypersonic aircraft’s dynamic trajectory.

Movement State	Time (s)	Forward Acceleration (m/s/s)	Pitch Rate (°/s)	Final Velocity (m/s)
Accelerative running	0–20	4.00	0	80.000
Accelerative taking-off	20–35	1.00	1	95.000
Accelerative climbing	35–75	2.00	0	175.00
Steady climbing	75–100	0	0	175.00
Change to level flight	100–115	0	−1	175.00
Steady level flight	115–145	0	0	175.00
Separation from carrier	145–150	0.68	0	178.40
Ignition of the rocket	150–153	10.0	0	208.40
Accelerative head-up	153–163	19.0	1	398.40
Accelerative climbing	163–250	19.0	0	2051.4
Change to level flight	250–260	0	−1	2051.4
Quick popup of X-43	260–265	19.0	0	2146.4
High acceleration of X-43	265–270	300	0	3646.4
Steady level flight	270–300	0	0	3646.4

**Table 3:** RMSEs of the velocity errors in one cycle.

RMSE of Velocity Errors	High-Precision IMU	Mid-Precision IMU	Low-Precision IMU
East	0.0150	0.0206	0.0805
North	0.0222	0.1241	0.5268
Up	0.0456	0.2267	0.6751
Calculated by Equation (28)	0.0601	0.1574	0.5724

**Table 4:** The phase errors calculated by the left-hand side of Equation (27) for the three kinds of IMUs.

IMUs	Calculated Phase Error (°)
High-precision	5.08280 (<15)
Mid-precision	11.7625 (<15)
Low-precision	34.1919 (>15)

**Table 5:** RMSEs of the deeply coupled system errors aided by the three kinds of IMUs.

RMSE of System Errors	High-Precision	Mid-Precision	Low-Precision
East-position (m)	1.9686	7.0726	407.51
North-position (m)	2.8281	6.9240	279.73
Up-position (m)	5.5569	11.482	210.98
East-velocity (m/s)	0.0740	0.4287	6.5085
North-velocity (m/s)	0.1307	0.4583	30.695
Up-velocity (m/s)	0.2115	0.8553	13.535

## References

[1] Zhang T., Niu X., Ban Y., Zhang H., Shi C., Liu J. (2015). Modeling and development of INS-aided PLLs in a GNSS/INS deeply-coupled hardware prototype for dynamic applications. Sensors.

[2] Liu G., Guo M., Zhang R., Peng Z., Luo S. (2013). MIMU precision’s influence on GNSS/MINS integrated navigation system performance by simulation analysis. J. Chin. Inert. Technol..

[3] Zeng Q., Meng Q., Liu J., Feng S., Wang H. (2016). Acquisition and loop control of ultra-tight INS/BeiDou integration system. Optik.

[4] Kirkko-Jaakkola M., Ruotsalainen L., Bhuiyan M.Z.H., Soderholm S., Thombre S., Kuusniemi H. Performance of a MEMS IMU Deeply Coupled with a GNSS Receiver under Jamming. Proceedings of the Ubiquitous Positioning Indoor Navigation and Location Based Service (UPINLBS).

[5] Adeel M., Chen X., Yu W., Ying R., Liu P. Performance Analysis of Deeply Coupled INS Assisted Multi-Carrier Vector Phase Lock Loop for High Dynamics. Proceedings of the 28th International Technical Meeting of The Satellite-Division-of-the-Institute-of-Navigation (ION GNSS+).

[6] Langer M., Trommer G.F. Multi GNSS constellation deeply coupled GNSS/INS integration for automotive application using a software defined GNSS receiver. Proceedings of the 2014 IEEE/ION Position, Location and Navigation Symposium (PLANS 2014).

[7] Slater C., Creaghan M., Lamce O. (2016). Six-Axis Monopropellant Propulsion System for Picosatellites.

[8] Chen Z. (2016). Analysis of the IMU precision’s influence on the loop of deeply-coupled GNSS/INS navigation system in high-dynamic environment. Optik.

[9] Bancroft J.B., Lachapelle G. Estimating MEMS Gyroscope G-Sensitivity Errors in foot mounted navigation. Proceedings of the Ubiquitous Positioning, Indoor Navigation, and Location Based Service (UPINLBS).

[10] Qin F., Zhan X., Zhan L. (2014). Performance assessment of a low-cost inertial measurement unit based ultra-tight global navigation satellite system/inertial navigation system integration for high dynamic applications. IET Radar Sonar Navig..

[11] Zeng Q., Chen W., Liu J., Wang H. (2017). An Improved Multi-Sensor Fusion Navigation Algorithm Based on the Factor Graph. Sensors.

[12] Wagner J.F. (2015). GNSS/INS integration: Still an attractive candidate for automatic landing systems?. GPS Solut..

[13] Xing L., Hang Y., Xiong Z., Liu J., Wan Z. (2016). Accurate Attitude Estimation Using ARS under Conditions of Vehicle Movement Based on Disturbance Acceleration Adaptive Estimation and Correction. Sensors.

[14] Xie G. (2009). Principles of GPS and Receiver Design.

[15] Xie F., Liu J., Li R., Jiang B., Qiao L. (2015). Performance analysis of a federated ultra-tight global positioning system/inertial navigation system integration algorithm in high dynamic environments. Proc. Inst. Mech. Eng. Part G.

